# Regional differences of individual and allocation efficiencies of health resources in China

**DOI:** 10.3389/fpubh.2023.1306148

**Published:** 2023-12-21

**Authors:** Qinpu Liu, Yuling Guo

**Affiliations:** ^1^Science and Technology Innovation Team of Health Tourism, Nanjing Xiaozhuang University, Nanjing, Jiangsu, China; ^2^Public Health Center, Nanjing Xiaozhuang University, Nanjing, Jiangsu, China

**Keywords:** public health service, multi-input multi-output, resources individual and allocation efficiency, Relative Productivity Proportion Weight, China

## Abstract

**Background:**

The existing health resources and services are difficult to meet the needs of rapid economic development and the aging population in China. This paper evaluates the regional differences of individual and allocation efficiencies of health resources in China to explore ways to change the current situation.

**Methods:**

The models of single-input single-output efficiency (SISOE), single-input multi-output efficiency (SIMOE), multi-input single-output efficiency (MISOE), and multi-input multi-output efficiency (MIMOE) were developed to calculate the individual and allocation efficiencies of health resources of China in this study.

**Results:**

It was found that the efficiencies of the number of health institutions (NHI) in the eastern and western regions of China were relatively close, with values of 0.61 and 0.59, respectively, significantly higher than 0.49 in the middle region. The efficiencies of the number of health personnel (NHP) in the eastern, middle, and western regions were closer, with values of 0.77, 0.75, and 0.79, respectively. The efficiencies of the number of health institution beds (NHIB) in the eastern and western regions were very close, with values of 0.79 and 0.78, respectively, while that in the middle region was 0.72. The efficiencies of the total health expenditure (THE) were 0.72, 0.76, and 0.79 in the east, middle, and western regions, respectively. The efficiencies of the number of diagnosis and treatment persons (NDTP) were 0.81, 0.70, and 0.71 in the eastern, middle, and western regions, respectively, while the efficiencies of the number of inpatients (NI) were 0.75, 0.79, and 0.81, respectively. The efficiencies of the utilization rate of beds (URB) and the average days of hospitalization (ADH) in the three regions were below 0.51. The health resources allocation efficiencies (HRAEs) were 0.86, 0.83, and 0.87 in the eastern, middle, and western regions, respectively.

**Conclusion:**

There were obvious regional differences in HRAE in China with the situation of “Middle Collapse.” The main direct reason for the low HRAE in the middle region was the lower efficiencies of NHI, NHIB, URB, and ADH. It revealed that there was relatively blind expansion of health institutions and beds with lower health service quality in the middle region. Governments should make strategic adjustments to public health resources and increase the investment in medical technology and manpower in the middle region. Hospitals in the eastern region should strengthen inter-regional medical and health technical cooperation with partners in the middle region by establishing a tele-medical network. The models of SISOE, SIMOE, MISOE, and MIMOE put forward in this study are simple, reasonable, and useful for resource efficiency analysis, which makes it convenient to adopt targeted measures to upgrade the efficiency of resource allocation. This study provides a new perspective and method to understand the mechanism of regional differences in China’s health resource allocation efficiency.

## 1 Introduction

Health resources are the basis for the development of health services, and the rationality of their allocation not only affects residents’ health levels but also plays an important role in the sustainable development of medical and health services (1). The equity and efficiency of the distribution of health resources are regarded as the main goals pursued by public health management and the basic principles advocated by the World Health Organization (2). With the development of China’s economy and society since carrying out the reform and opening up policy in 1978, China’s investment in health resources is increasing, medical and health services have made remarkable achievements, and people’s health has significantly improved. However, due to the different levels of development in the east, west, and middle regions, the imbalance in the investment of health resources among regions still exists. Since 2009, China implemented the new medical and health system reform, making the health resource allocation structure greatly improved, but the allocation efficiency of health resources is still at a low level (3). The contradiction between the insufficient supply of health services and the growing demand is still prominent (4, 5). The COVID-19 international public health emergency that broke out at the end of 2019 posed a serious challenge to the medical and health service capacity and regional equity of China (6). At present, while paying attention to health resource investment, it is necessary to optimize the investment structure, improve the efficiency of health resource utilization, and enhance the fairness of health services in various regions of the country. This requires an analysis of the efficiency of health resource allocation in the eastern, middle, and western regions. It is one of the important topics in the research of China’s health service sustainability (7).

The research methods for health resource allocation efficiency mainly include two types: one is the traditional parametric method based on the production frontier theory—Stochastic Frontier Analysis (SFA); the other is the non-parametric method of data envelopment analysis (DEA) (8, 9). SFA is an effective method for cost efficiency measurement under the “multi-input, single-output” condition. DEA meets the requirements of “multi-input and multi-output” for measurements of the technical efficiency (TE) of resource allocation (10). Varabyova and Schreyogg (11) used the SFA method to calculate the hospital operation efficiency in OECD countries and believed that the *per capita* healthcare expenditure had a positive impact on hospital technical efficiency. Li (12) also used the SFA method to evaluate the efficiency of the medical security system in OECD countries from 1996 to 2009 and found that their efficiency values were less than one on average, meaning that they were inefficient. Zhao and Zhang used the SFA method to calculate the cost efficiency of health resource allocation of 31 provinces in China in 2014. The results showed that the average cost efficiency value was 0.423. Except for nine provinces, 22 provinces operated in low or medium efficiency (13). Retzlaffrobert et al. (14) used the DEA method to calculate the efficiency of medical insurance resources in OECD countries in 2000 and believed that the efficiencies of medical security systems in countries such as Japan, Norway, Canada, and Sweden were higher, all of which were with good health outcomes. Using DEA, Mark et al. (15) examined the technical efficiency of 226 medical, surgical, and medical-surgical nursing units in 118 acute care hospitals randomly selected in the United States and found that less than half of nursing units operated at the optimal level of efficiency. Nayar et al. (16) conducted a DEA analysis for 371 nationally representative acute care hospitals in the United States in 2008 and found that incorporating quality into the DEA models would be a better reflection of the hospital product. The input-oriented CCR-DEA model was used to study the efficiency of several hospital health centers in Greece, and excellent performance was found for the units, additionally providing preventive medical services (17, 18). The efficiency of intensive care units was valued with the BCC-DEA model in Iran, and five hospitals were identified as efficient in technical, managerial, and scale performance (18, 19). Xu et al. (20) selected the relevant indicator data of 31 provinces in China in 2018, using the three-stage DEA model to investigate the efficiency of health resource allocation, and found out that after removing the impact of environmental factors and random noise, the comprehensive technical efficiency of the national health resource allocation increased from 0.859 to 0.893, and the health resource allocation efficiency in the middle and eastern regions of China was better than that in the western region. Peng et al. (21) used the super-efficiency DEA window to compare the efficiency of health resource allocation in the eastern, middle, and western regions of China, and the results showed that from 2009 to 2016, the annual average of super-efficiency of the health resources allocation in the three regions was 1.017, 0.957, and 1.004, respectively. Chen and Cao (22) studied the efficiency of health resource allocation in 31 provinces of China from 2013 to 2016 with the three-stage DEA model removing the interference of environmental factors and random factors, and they found that the efficiencies of health resource allocation in 4 years were 0.545, 0.570, 0.574, and 0.572, respectively, and the efficiency of the eastern region was the highest, followed by the western and the middle regions.

The methods illustrated above for studying the allocation efficiency of health resources are relatively complex, and they fail to express the efficiency of each individual resource under the condition of “multi-input and multi-output.” Based on the concept of “Relative Productivity Proportion Weight (RPPW)” (23–25), which means that the weight of one index’s relative productivity is equal to its proportion in the sum of relative productivities of all indexes involved, this paper puts forward new models simultaneously to investigate regional individual and allocation efficiencies of health resources in China under the condition of multi-input and multi-output. It tries to improve the measurement method of health resource efficiency and provides China’s health institutions and administrative departments with references that would help formulate targeted policies to optimize the allocation of health resources and promote the sustainable development of health services in China.

The main contribution of this study lies in the following innovations:

1. This study found that there were obvious regional differences in health resource allocation efficiency in China, with the situation of “Middle Collapse” and revealed the direct reasons for that in view of individual health resource efficiency. It provides a new perspective and method to understand the mechanism of regional differences in China’s health resource allocation efficiency.
2. Compared with DEA, the models put forward in this study are simple and practical, are convenient to adopt targeted measures to upgrade the efficiency of health resource allocation, and can also be used for other field efficiency studies.

## 2 Materials and methods

### 2.1 Data sources and region division

The data used in this study are from the China Health Statistics Yearbook (2021) prepared by the National Health Commission of the People’s Republic of China (26), and the National Data published on the website of the National Bureau of Statistics of the People’s Republic of China (27). China’s 31 provinces (including autonomous regions and municipalities directly under the Central Government), i.e., 31 DMUs, are geographically divided into three sections: the eastern, middle, and western regions. The eastern region includes 11 provinces of Beijing, Tianjin, Hebei, Liaoning, Shandong, Shanghai, Jiangsu, Zhejiang, Fujian, Guangdong, and Hainan; the middle region includes 8 provinces of Jilin, Heilongjiang, Shanxi, Henan, Anhui, Hubei, Hunan, and Jiangxi; the western region includes 12 provinces of Neimeng (Inner Mongolia), Ningxia, Shaanxi, Gansu, Qinghai, Chongqing, Sichuan, Guizhou, Yunnan, Guangxi, Xizang (Tibet), and Xinjiang. Because the data for Hong Kong, Macao, and Taiwan are not available, they are not studied in this paper.

### 2.2 Indicators of health efficiency

The indicators of evaluating health resource efficiency include two parts: health resource input indicators and health service output indicators. Considering representation, stability, and independence, property, manpower, and finance investments are deemed to be important input variables in the delivery of health services. Consistent with most previous relevant studies (7, 28), the number of health institutions (NHI), the number of health institution beds (NHIB), the number of health personnel (NHP), and the total health expenditure (THE) were selected as health resource input indicators in this paper. NHI and NHIB, NHP, and THE represent property, manpower, and finance, respectively. Because the health services in health institutions are mainly for outpatients and inpatients, with quantitative and qualitative features, quantity and quality indicators combined can better reflect the relationship between resource input and service output. Therefore the number of diagnosis and treatment persons (NDTP) in various medical and health institutions, the number of inpatients (NI), the utilization rate of beds (URB; the ratio of the total number of bed days actually occupied to the total number of bed days actually opened), and the average days of hospitalization (ADH) were selected as health service output indicators. The first three outputs are quantitative indicators, while ADH belongs to the qualitative indicator. Different from the other seven indicators, ADH is negative because the shorter the ADH, the higher the efficiency of health services (29). It is necessary to make the polarity of all variables consistent before conducting data calculation. The reciprocal method (1/X) was used to adjust the original data of ADH to be positive (30). Although the relationship between ADH and its reciprocal is non-linear, the correlation coefficient between the two sets of values in this study is 0.995, indicating that the reciprocal method is appropriate here.

### 2.3 Models

#### 2.3.1 Raw data processing by maximum normalization

The health resource efficiency in this study is the same as the technical efficiency in the DEA method, both of which are relatively efficient. It is necessary to standardize the data of all indicators to eliminate the dimensional differences, making the values of indicators within the range of zero to one. We therefore standardize the original data of health resource inputs and service outputs by their maximum values. The formula is given as follows:


(1) $$N_{i}=\frac{x_{i}}{x_{i}\left(\max\right)}\;\left(i=1,\;2,\;\cdots ,\;n\right)$$

(2) $$N_{p}=\frac{x_{p}}{x_{p}\left(\max\right)}\;\left(p=1,\;2,\;\cdots ,\;k\right)$$

where *N_i_* and *N_p_* in formulas 1, 2 represent the normalized values of input indicators and output indicators by each maximum, respectively; *x_i_* and *x_p_* represent the original values of input and output indicators, respectively; *x_i_*(max) and *x_p_*(max) represent the maximum of the original values of the input and output indicators, respectively; *n* represents the number of input indicators; *k* represents the number of output indicators.

#### 2.3.2 Single-input single-output efficiency (SISOE)

The SISOE refers to the ratio of each output value to each input value. It is the basis for measuring the efficiencies of single-input multi-output, multi-input single-output, and multi-input multi-output. The formula of SISOE is given as follows:


(3) $$E_{s-s}=\frac{N_{p}}{N_{i}}\;\left(s-s=1,\;2,\;\cdots ,\;n\cdot k\right)$$

where *E_s-s_* represents the SISOE; the subscript *s-s* of *E_s-s_* means from single input to single output. There are four health resource input indicators and four health service output indicators in this study; each input indicator relates to four output indicators, and each output indicator relates to four input indicators; therefore, 16 efficiency values of SISOE in one DMU will be obtained.

The SISOE will have positive values greater or less than one. In order to define the values of SISOE between zero and one, they must be standardized by the maximum. The formula is given as follows:


(4) $$E_{s-s}^{'}=\frac{E_{s-s}}{E_{s-s}\left(\max\right)}\;\left(s-s=1,2,\cdots ,n\cdot k\right)$$

where *E’_s-s_* is the standardized value of *E_s-s_* by the maximum. *E_s-s_* (max) is the maximum value of *E_s-s_*.

#### 2.3.3 Single-input multi-output efficiency (SIMOE) and multi-input single-output efficiency (MISOE)

Under the condition of multi-input and multi-output, each input indicator relates to all multi-output indicators and so does each output indicator, so we can get any one of SIMOEs, which is called individual efficiency, or any one of MISOEs based on the SISOEs. Because the SIMOE is the efficiency of a single input corresponding to multi-outputs in the perspective of input, the measurement of one input SIMOE is several related SISOEs multiplied by their proportion in all these SISOEs, respectively, then added together and raised to the power of θ. This is called the “Relative Productivity Proportion Weight” method (23–25). The empirical formula is given as follows:


(5) $$E_{s-m}={\left(\frac{\sum E_{s-s}^{'2}}{\sum E_{s-s}^{'}}\right)}^{\theta }\;\left(s-m=1,2,\cdots ,n\right)$$

Where *E_s-m_* represents the SIMOE based on SISOE in view of one input indicator, the subscript *s-m* of *E_s-m_* means from single input to multiple outputs, and *θ* is called the adjustment coefficient with the values of one to zero; it adjusts the value of *E_s-m_*. The smaller the value of *θ*, the higher the values of *E_s-m_* and the smaller the difference of *E_s-m_* values among DMUs. Usually, the value of *θ* could be taken as 0.5. *n* represents the number of inputs. There are four health service outputs in this study, and each input has four related SISOEs, so the first one of four health resource input SIMOEs, for example, can be obtained with the following formula:


(6) $$E_{s-m}={\left(\frac{\begin{array}{l} E_{s1-s1}^{'}\cdot E_{s1-s1}^{'}+E_{s1-s2}^{'}\cdot E_{s1-s2}^{'}+\\ E_{s1-s3}^{'}\cdot E_{s1-s3}^{'}+E_{s1-s4}^{'}\cdot E_{s1-s4}^{'} \end{array}}{E_{s1-s1}^{'}+E_{s1-s2}^{'}+E_{s1-s3}^{'}+E_{s1-s4}^{'}}\right)}^{\frac{1}{2}}$$

Similarly, each output indicator has four related input indicators. If taking SISOE as the measurement basis from the perspective of output, one of the health service output MISOEs can be obtained. The formula is given as follows:


(7) $$E_{m-s}={\left(\frac{\sum E_{s-s}^{'2}}{\sum E_{s-s}^{'}}\right)}^{\theta }\;\left(m-s=1,2,\cdots ,k\right)$$

where *E_m-s_* represents the health service output MISOE, and the subscript *m-s* of *E_m-s_* means from multiple inputs to a single output. *k* represents the number of outputs; *k* = 4 in this study. It should be noted that the *E’ s-s* in formula 5 is not the same as the *E’ s-s* in formula 7.

#### 2.3.4 Multi-input multi-output efficiency (MIMOE)

MIMOE indicates the ability of various resources to reach the best proportion in order to achieve the maximum output (31). It is the integrated value of SIMOEs (or MISOEs) that can be obtained by the weighted average of all SIMOEs (or MISOEs). The health resource allocation efficiency (HRAE) belongs to multi-input multi-output efficiency. The formula for the measurement of HRAE (or MIMOE) is given as follows:


(8) $$E_{m-m}={\left(\frac{\sum E_{s-m}^{2}}{\sum E_{s-m}}\right)}^{\theta }\;\left(s-m=1,2,\cdots ,n\right)$$

or


(9) $$E_{m-m}={\left(\frac{\sum E_{m-s}^{2}}{\sum E_{m-s}}\right)}^{\theta }\;\left(\;m-s=1,2,\cdots ,k\right)$$

where *E_m-m_* represents MIMOE or HRAE; the subscript *m-m* of *E_m-m_* means from multiple inputs to multiple outputs. It is noted that the value of *E_m-m_* in formula 8 will be slightly different from that in formula 9.

The values of *E_s-m_*, *E_m-s_*, and *E_m-m_* range from zero to one. Referring to the classification of efficiency in relevant reference (24, 32) and the actual distribution of health resource efficiency in China, the values of health resource efficiency in the ranges of <0.65, 0.65–0.75, 0.75–0.85, 0.85–0.95, and ≥ 0.95 are classified as very low, low, medium, high, and very high efficiencies, respectively.

## 3 Results

### 3.1 Regional differences of individual health resource efficiency in China

**Figure 1:**
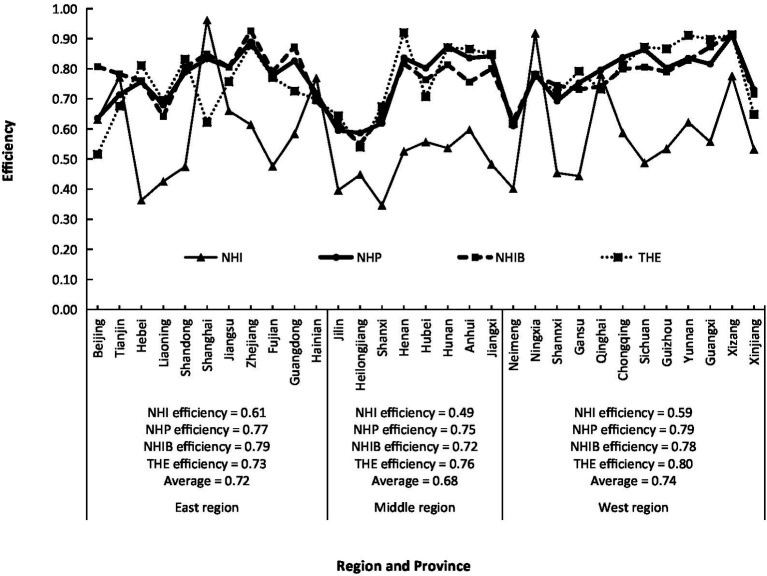
Regional differences of efficiencies of the number of health institutions (NHI), the number of health personnel (NHP), the number of health institutions beds (NHIB), the total health expenditure (THE) in China.

According to formula 5, the individual health resource efficiency of four indicators (NHI, NHP, NHIB, and THE) of China in 2020 was calculated (Figure 1). In general, except for Beijing, Tianjin, Shanghai, and Ningxia, the efficiencies of NHI in 27 provinces were significantly lower than those of NHP, NHIB, and THE, indicating that there were too many health institutions to be used efficiently. Besides Beijing, Tianjin, and Shanghai, the input efficiency values of NHP, NHIB, and THE of each province were basically similar. For example, the efficiencies of NHP, NHIB, and THE of Hebei Province are 0.76, 0.76, and 0.78, respectively, as well as 0.59, 0.62, and 0.61 for Jilin Province, and 0.91, 0.91, and 0.91 for Xizang. However, there were obvious differences in four health resource efficiencies among three regions and provinces. The efficiencies of NHI in the eastern and western regions were relatively close, with 0.61 and 0.59, respectively, significantly higher than 0.49 in the middle region, and all of them belonged to very low efficiency. Among the 31 provinces, Shanghai and Ningxia had the highest efficiency of NHI, with values of 0.96 and 0.92, respectively, both of which were at the level of very high efficiency and high efficiency, respectively, while Shanxi and Hebei had the lowest efficiency, with the values of 0.35 and 0.36, respectively, both of which belonged to very low efficiency. The difference in NHP efficiency in the east, middle, and west was smaller, with values of 0.77, 0.75, and 0.79, respectively, all of which were at the middle-efficiency level. The efficiencies of Xizang and Zhejiang were the highest, with values of 0.91 and 0.89, respectively, while Jilin, Heilongjiang, and Neimeng were the lowest, with values of 0.59, 0.59, and 0.61, respectively. The efficiency of NHIB in the eastern and western regions was very close, with values of 0.79 and 0.78, respectively, both of which belonged to medium efficiency, while that in the middle region was 0.72, belonging to low efficiency. Zhejiang and Xizang had the highest values of 0.93 and 0.91, respectively, belonging to high efficiency; but Liaoning, Jilin, Neimeng, and Heilongjiang had the lowest values of 0.64, 0.62, 0.63, and 0.55, respectively, at very low efficiency, most of which lies in the Northeast of China. The efficiencies of THE in the western and middle regions were at medium levels with values of 0.80 and 0.76, respectively, while the eastern region had a low efficiency of 0.73. Xizang, Yunnan, and Henan had the highest efficiencies of THE with values of 0.91, 0.91, and 0.92, respectively, all of which belonged to high-efficiency levels, but Beijing and Heilongjiang had the lowest efficiencies of 0.52 and 0.54, respectively, both of which belonged to very low efficiency.

### 3.2 Regional differences of individual health services efficiency in China

**Figure 2:**
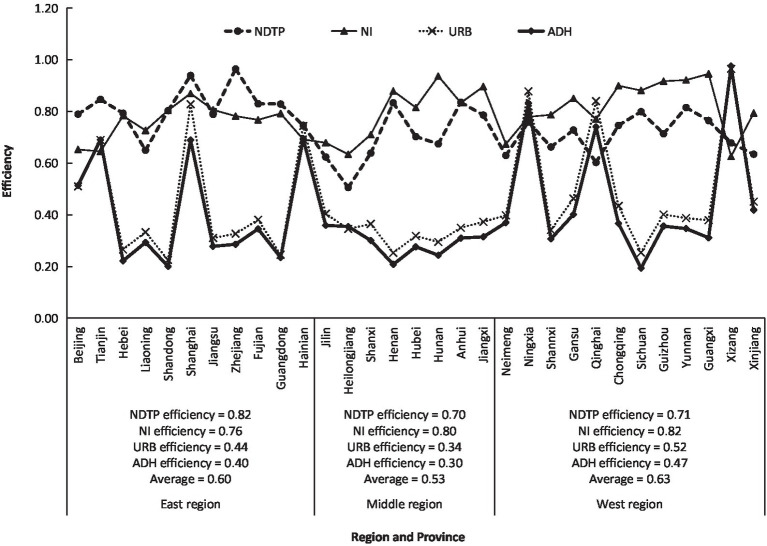
Regional differences of efficiencies of the number of diagnosis and treatment persons (NDTP), the number of inpatients (NI), the utilization rate of beds (URB), and the average days of hospitalization (ADH) in China.

In order to examine the efficiency of health resource input from the perspective of health service output, the individual efficiency of four health service outputs of China in 2020 was calculated according to formula 7 (Figure 2). It can be seen from Figure 2 that the efficiency of NDTP had a larger difference from that of NI in and among provinces. The efficiency of NDTP in the east was at a medium level, those in the middle and west were low, while the efficiency of NI was at a medium level in the east, middle, and west. Zhejiang and Shanghai had the highest efficiency of NDTP, with values of 0.96 and 0.94, belonging to very high efficiency and high efficiency, respectively, while Heilongjiang, Jilin, Neimeng, Qinghai, and Xinjiang had the lowest efficiency, with the values of 0.51, 0.62, 0.63, 0.60, and 0.63, respectively, all of which belonged to very low efficiency. Guangxi, Hunan, Yunnan, and Guizhou had the highest efficiencies of NI, with the values of 0.94, 0.94, 0.92, and 0.92, respectively, all of them belonging to high efficiency, but Tianjin, Heilongjiang, and Xizang had the lowest efficiency, with the values of 0.64, 0.63, and 0.63, respectively, all of them belonging to very low efficiency.

It is interesting that the efficiencies of URB and ADH in the eastern, middle, and western regions were below 0.52, all belonging to very low efficiency. The efficiencies of URB and ADH within each province were close to each other, and their regional change trends were highly consistent, with a correlation coefficient of 0.99. Except for six provinces of Tianjin, Shanghai, Hainan, Ningxia, Qinghai, and Xizang, whose efficiencies of URB and ADH were above 0.65, the other 25 provinces had very low efficiencies. Xizang had the highest efficiencies of URB and ADH, with values of 0.96 and 0.97, respectively, followed by Ningxia, with values of 0.88 and 0.83, respectively, while Shandong and Sichuan had the lowest efficiencies of URB and ADH, with values of 0.23 and 0.20 and 0.25 and 0.19, respectively.

### 3.3 Regional differences of HRAE in China

**Figure 3:**
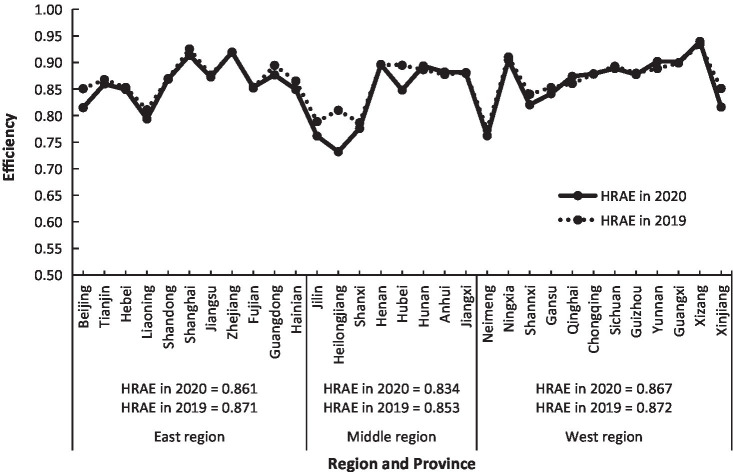
Regional difference of HRAEs of China in 2020 and 2019.

According to formula 8, the MIMOE, i.e., HRAE of China in 2020, was calculated (Figure 3). On average, China’s HRAE in 2020 was 0.85, belonging to high efficiency. There were obvious regional differences in HRAE in China. The eastern and western regions had 0.86 and 0.87, respectively, both of them belonging to the high efficiency, and the middle region had 0.83 at the middle-efficiency level. It could be seen that China’s HRAE was then in the situation of “Middle Collapse” (21), meaning that the health development in the middle region lagged behind the eastern and western regions. Among the 31 provinces, the range of HRAE was between 0.73 to 0.94, from low efficiency to high efficiency. The highest values were 0.94, 0.92, and 0.91 for Xizang, Zhejiang, and Shanghai, respectively, the lowest values were 0.73, 0.76, and 0.76 for Heilongjiang, Jilin, and Neimeng, respectively. There were different reasons for these provinces with big different values of HRAE. Xizang had the highest HRAE due to the higher efficiency of ADH, URB, NHP, NHIB, and THE, while Shanghai due to the higher efficiency of ADH, URB, and NHI, and Zhejiang due to the higher efficiency of NDTP, NHP, NHIB, and THE. The lowest HRAE in Heilongjiang, Jilin, and Neimeng results from the fact that their individual efficiency is relatively lower. According to Figures 1, 2, the direct reason for lower HRAE in the middle region resulted mainly from low efficiencies of NHI and NHIB and URB and ADH. It indicates that there are relatively blind expansion of health institutes, insufficient utilization of beds, and lower quality of health service in the middle region. The indirect reason for that might be due to the shortage of technology, equipment, and high-level professional technicians, especially in less populated areas like Jilin and Heilongjiang provinces with very low allocation efficiencies of health resources. The higher efficiency of resource allocation in the eastern region is attributed to the fact that the eastern region has relatively excellent health technicians, advanced medical equipment, and high-quality diagnosis and treatment protocols, which attract more people to go there to see doctors, so it has high diagnosis and treatment efficiency and high bed utilization efficiency. The higher allocation efficiency in the western region lies in the reasonable supply structure and proper operation scale in the process of transforming health resources into health services (33).

In order to check the stability of regional differences of HRAE in China and the impact of the COVID-19 pandemic on it, the regional difference of HRAE of China in 2019 was calculated (Figure 3). The result showed that most provinces had the same or similar values of HRAE in 2020 compared with those in 2019, except for Beijing, Heilongjiang, Hubei, and Xinjiang. It indicates that there is little change in the regional difference pattern of HRAE although the COVID-19 pandemic hit the country. But it is obvious that averages of HRAEs in the three regions in 2020 were a little lower than those in 2019 because most provinces enhanced more inputs of money, personnel, and materials to deal with the pandemic situation of COVID-19, except for Xizang, Qinghai, and Yunan all of which were located in the remote area with a little higher HRAE.

## 4 Discussion

RPPW is an objective valuation method to determine the weights of the indexes based on their own values. It not only reduces the subjective influence of decision-makers and improves objectivity but also takes into account the importance and mutual influence of indexes. If one index value has a larger proportion in all, it shows that this index plays a more important and effective role than others in determining the difference among all decision-making units (DMUs). Moreover, using the RPPW method to obtain resource allocation efficiency can ensure the allocation efficiency reaching the maximum value of one when all the individual resource efficiencies reach the maximum value of one. Based on RPPW, the models of SISOE, SIMOE, MISOE, and MIMOE developed in this study are simple and practical. They are easy to use without the help of special computer software, so their economic meaning is obvious. Using SIMOE and MISOE, we can obtain the individual health resource input efficiency and individual health service output efficiency, respectively. Therefore we can see which single-input efficiency or single-output efficiency plays a major role in the MIMOE or HRAE. It is convenient for us to take targeted measures to improve the allocation efficiency of health resources.

**Figure 4:**
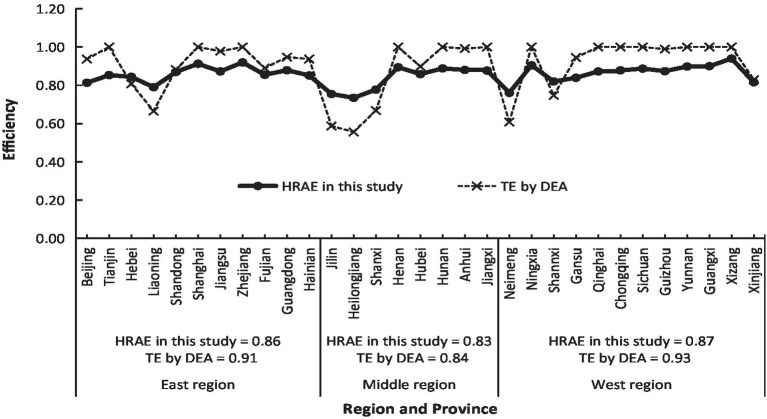
Regional differences of health resource allocation efficiency (HRAE) of China in this study and technical efficiency (TE) by data envelopment analysis (DEA) method.

In order to prove the feasibility of the MIMOE model, the DEA method was used to process the same input and output data of China in 2020 used in this study and calculate the technical efficiency (TE). Comparing the HRAE in this study with the TE obtained by DEA, it was found that the results from the two methods were very similar (Figure 4), with averages of 0.86 and 0.90, respectively, and a relative error of 4.4%. Moreover, the trend of regional difference of HRAE in this study was basically consistent with that of TE by the DEA method, with a correlation coefficient of 0.916, indicating that the method used in this study was reasonable. It was noted that compared with that in DEA, the result of this study adjusted the regional difference to be less. Especially, 13 provinces (Tianjin, Shanghai, Zhejiang, Henan, Hunan, Jiangxi, Ningxia, Qinghai, Chongqing, Sichuan, Yunnan, Guangxi, and Xizang) with technical efficiency of one in DEA analysis were also distinguished. This is like the method of the Super-efficiency DEA model which is an improvement on the traditional DEA for distinguishing some DMUs with the same TE of one (34, 35).

**Figure 5:**
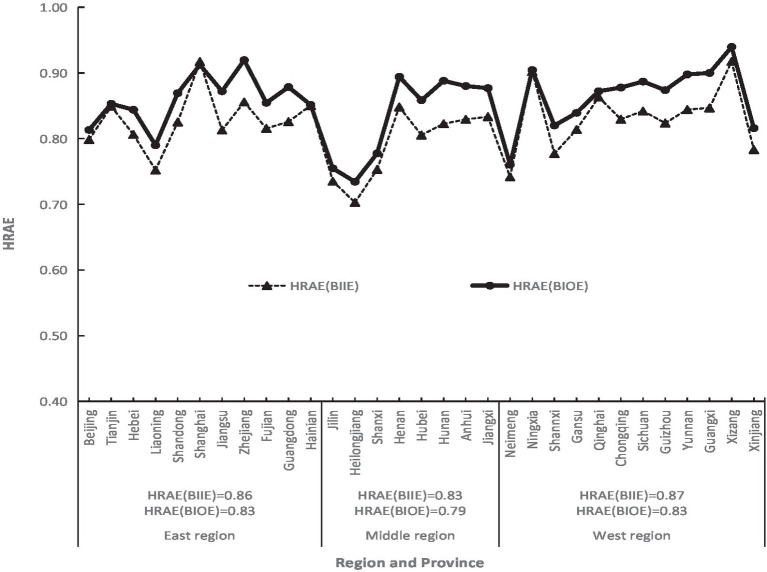
Comparison of health resource allocation efficiency (HRAE) based on individual input efficiency (BIIE) with that based on individual output efficiency (BIOE) of China in 2020.

The HRAE of China’s 31 provinces in 2020 can be obtained not only by formulas 5, 8 based on individual input efficiency but also by formulas 7, 9 based on individual output efficiency. The correlation coefficient of the two results is 0.924, showing a high correlation (Figure 5). Their average values are 0.82 and 0.85, respectively, with a relative error of 4%. This is just like the DEA method, with the output-oriented model and the input-oriented model, which can be used to calculate the TE (36), and the results of the two ways would also be different.

There are obvious regional differences of HRAE in China with an average of 0.85. In terms of the three regions, the efficiency of health resource allocation in the middle region is significantly lower than those in the eastern and western regions. The conclusion of this current study is different from that of Hu and Chen (37) who used the DEA method to study the efficiency of China’s health resource allocation in 2020. They found that the efficiency of the eastern region was the highest, and the western region was the lowest. This may be due to the differences in indicator numbers and data processing methods. They selected four input indicators and three output indicators, including ADH. Hu and Chen’s indicators were measured in both *per capita* values and total absolute ones and did not explain how to deal with the ADH. We believe that efficiency is the comparison of input with output in the same DMU. The values of indicators such as NHI, NHP, NHIB, THE, NDTP, and NI were either absolute values or *per capita* so that they could be compared.

Although this study has attempted to select the justifiable indicators and used some new models to evaluate the regional difference in health resource allocation efficiency in China, there are still some limitations. First, the indicator of health personnel was not further subdivided into doctors, nurses, and others, and the health institutions were not further subdivided into hospitals, grassroots health institutions, professional public health institutions, and others. Future studies could employ additional indicators for detailed analysis. Second, the models of formulas 5–9 are empirical ones. Although the result of this study is in high accordance with that of the DEA method, we now have no theoretical reason to explain it. Third, different selections of power θ in the models will affect the results. There is an interesting fact that the value of θ in formula 8 affects the mean and variance of HRAE, as well as the correlation coefficient between the HREA in this study and TE by the DEA method. The smaller the value of θ, the smaller the variance of HRAE, the closer to one the mean of HRAE, and the higher the correlation coefficients between HRAE and TE. How to select the value of θ more appropriately needs to be studied in the future.

## 5 Policy recommendation

China’s HRAE is not very high in 2020 with great differences between regions and provinces. The low HRAE in the middle region of China may be due to the blind increase of medical institutions, resulting in their insufficient utilization (21). With more medical institutions, there are more beds and lower bed utilization rates. According to the official statistics, grassroots health institutions account for over 90% of health institutions in China. Among the three regions, the middle region has the highest proportion of grassroots health institutions, reaching over 95% in 2019 and 2020. The overall efficiency of health resource allocation in grassroots health institutions in China is low, and their development is uneven. In 2019, the average technical efficiency of health resource allocation in the grassroots health institutions in China was at a low value of 0.73 (38). The main reason is that the governments put less and unreasonable investments in grassroots health institutions, resulting in poor equipment, fewer technical personnel, and poor service quality. The residents prefer going to large hospitals to see doctors than to the grassroots health institutions, giving rise to the waste of resources therein. Therefore, to solve the problems of lower efficiency and large regional differences in the allocation of health resources in China, we should start from the grassroots health institutions.

Governments at all levels should play the leading role in the supply of health resources inputs and services outputs. There are some suggestions given below: First, the central government should strengthen policy support, optimize the regional economic structure, and make strategic adjustments to public health resources. Local governments in the middle region should formulate policies from the aspects of economic development and population, optimize the allocation of health resources, and promote their rational distribution. The second is to increase the investment in medical technology and manpower in the middle region, actively adjust the structure of medical personnel, optimize the talent allocation structure, and upgrade the health institution management ability to provide local residents with high-quality health services. It is urgent to vigorously develop medical technology and projects, accelerate the training of a large number of qualified general practitioners, and strengthen the construction of the primary medical and health service system in the low-efficiency areas. For example, the local governments should promote the contracted services of family doctors and establish a hierarchical diagnosis and treatment system to meet the basic health and medical service needs of all residents. Third, the public welfare attributes of public hospitals should be strengthened to reduce the profit-oriented demand by determining reasonable salary levels of public hospitals and improving the salary and working conditions of healthcare workers at the grassroots level, especially in remote rural areas. Local governments should attract talent as much as possible to enlarge the grassroots medical professional team so as to realize the reasonable and effective allocation of health resources and the equalization of basic public health services in all provinces (8, 39). Fourth, the authorities should strengthen the supervision of medical and health undertakings, and deeply investigate the hospital management methods under the new situation of medical system reform so as to improve the medical quality and service level. Different regional environmental conditions have a great impact on the HRAE; governments at all levels should formulate reform plans according to the efficiency types of regions, and the evaluation indicators on hospitals should also be adjusted according to the regional development and environmental factors.

In addition, hospitals in the eastern region should strengthen inter-regional medical and health technical cooperation with partners in the middle and western regions by establishing a tele-medical network. Within regions and provinces, big hospitals and grassroots health institutions should strengthen the co-construction and share medical information. Some information technologies should be used to promote the horizontal and vertical flow of medical resources and improve the accessibility of high-quality medical resources and the overall efficiency of medical services. Different hospitals should promote the trans-regional specialty alliance and combine the same departments of inter-regional medical institutions to form a horizontal consortium. Large hospitals should provide training and technical guidance to doctors at the grassroots levels to provide high-quality and standardized services for their patients. Hospital managers should continuously improve the management system in the hospital, innovate the management form, and focus on strengthening the relatively scarce resource allocation, such as mental health, older adult care, pediatrics, rehabilitation, and family beds. All hospitals should strengthen the quality management of medical services, improve work efficiency, and provide residents with high-quality and satisfactory medical and health services according to regional characteristics.

## 6 Conclusion

The present study proposed some models to investigate regional individual and allocation efficiency of health resources in China under the condition of multi-input and multi-output based on the most recent available data from official publications.

It was found that there are obvious regional differences in HRAE in China, presenting a Middle Collapse” state. The HRAE in the eastern and western regions were 0.86 and 0.87, respectively, which belonged to the high-efficiency level, and the middle region was 0.83, belonging to the middle efficiency. This study also revealed that the direct reasons for the low HRAE in the middle region are mainly from the low efficiency of NHI, NHIB, URB, and ADH with values of 0.49, 0.72, 0.34, and 0.30, respectively. These findings show that there are relatively blind expansions of health institutions and beds in the middle region, with a lower quality of health service. The governments should make strategic adjustments to public health resources and increase the investment in medical technology and manpower in the middle region. Hospitals in the eastern region should strengthen inter-regional medical and health technical cooperation with partners in the middle region by establishing a tele-medical network to improve the accessibility of high-quality medical resources and the overall medical services efficiency in the middle region.

The individual and allocation efficiencies of health resources calculated by the models in this study reflect well the situation of China’s regional resources allocation. Compared with DEA, the models of SISOE, SIMOE, MISOE, and MIMOE are simple, practical, and easy to use for individual and allocation efficiency analyzes of health resources. They can also be used for other field efficiency studies. Because the allocation efficiency is composed of individual resource efficiency, it is convenient to identify the low individual efficiency and take targeted measures to improve the allocation efficiency of health resources. This study provides a new perspective and method to analyze the regional difference in health resource efficiency in China.

## Data availability statement

Publicly available datasets were analyzed in this study. This data can be found at: http://www.nhc.gov.cn/.

## Author contributions

QL: Writing – original draft, Methodology, Writing – review & editing. YG: Data curation, Writing – review & editing.
